# Seroincidence of HIV and prevalence of transmitted drug resistance of HIV-1 strains among persons seeking voluntary counselling and testing in Taiwan

**DOI:** 10.7448/IAS.17.4.19758

**Published:** 2014-11-02

**Authors:** Wen-Chun Liu, Lan-Hsin Chang, Pei-Ying Wu, Yu-Zhen Luo, Cheng-Hsin Wu, Yi-Ching Su, Chung-Chi Lai, Sui-Yuan Chang, Chien-Ching Hung

**Affiliations:** 1 Internal Medicine, National Taiwan University Hospital, Taipei City, Taiwan; 2 Center for Infection Control, National Taiwan University Hospital, Taipei City, Taiwan; 3 Internal Medicine, Kaohsiung Medical University Hospital, Kaohsiung City, Taiwan; 4 Clinical Laboratory Sciences and Medical Biotechno, National Taiwan University College of Medicine, Taipei City, Taiwan

## Abstract

**Introduction:**

The total case number of persons who are newly diagnosed with HIV continues to increase in Taiwan and men who have sex with men (MSM) have re-emerged as the leading risk group for HIV transmission. In this study, we aimed to estimate the incidence rate of HIV infection among those individuals who sought voluntary counselling and testing (VCT) service at a university hospital.

**Methods:**

Between 1 April, 2006 and 31 December, 2013, 18,246 tests for HIV antibody were performed among 12143 individuals at the VCT service. A total of 2157 individuals who tested negative for anti-HIV antibody had subsequent follow-up tests at the same VCT service, which composed the study population for estimation of incidence rate of recent HIV infection. The BED assays were used to identify recent HIV infections that occurred within the previous six months before seeking VCT service.

**Results:**

During the 6.5-year study period, 647 individuals were diagnosed as being HIV-positive, with an overall HIV seroprevalence of 3.55% (95% CI 3.27–3.82). The overall incidence rate of HIV infection was estimated 4.13 per 100 person-years of follow-up (95% CI 3.67–4.69 per 100 person-years of follow-up). MSM had an estimated 10-fold higher seroprevalence and seroincidence of HIV than heterosexuals. Of 647 clients testing positive for HIV, 603 clients were MSM (93.2%) and 477 patients (70.8%) subsequently sought HIV care at the hospital; 226 (47.4%) were diagnosed as having recent HIV infections by the BED assay, while 244 (51.2%) long-term infection and 7 without data by the BED assay. Of those patients, 173 (75.6%) and 178 patients (73.0%) with recent HIV infection and long-term infection had data of transmitted drug resistance mutations, respectively. The prevalence of transmitted drug resistance mutations to any class of antiretroviral therapy was 9.0% and 10.6% (p=0.68), respectively, of the HIV-1 strains from the patients with recent HIV infection and long-term infection, respectively.

**Conclusions:**

The seroincidence rate of HIV among persons seeking VCT was estimated 4.13 per 100 person-years of follow-up. The prevalence of transmitted drug resistance to any class of antiretroviral agents was similar between those who were recently infected with HIV and those who had long-term infection in Taiwan.

